# Complex tibial fractures are associated with lower social classes and predict early exit from employment and worse patient-reported QOL: a prospective observational study of 46 complex tibial fractures treated with a ring fixator

**DOI:** 10.1007/s11751-017-0301-y

**Published:** 2017-11-04

**Authors:** Rasmus Elsoe, Peter Larsen, Juozas Petruskevicius, Søren Kold

**Affiliations:** 1 Department of Orthopaedic Surgery, Aalborg University Hospital, Aalborg University, 18-22 Hobrovej, 9000 Aalborg, Denmark; 2 Department of Occupational Therapy and Physiotherapy, Aalborg University Hospital, Aalborg, Denmark

**Keywords:** Ilizarov, Ring fixator, Complex fracture tibial bone, Plateau fracture, Pilon fracture, Outcome

## Abstract

The long-term outcomes following complex fractures of the tibia are reported to carry a risk of knee pain, malalignment, articular injury and post-traumatic osteoarthritis. The main objective of this study was to account for the patient-reported quality of life (QOL) 12 months after ring fixator removal in patients with a complex tibial fracture. Secondary objectives included a review of the socio-economic characteristics of the patient group and the rate of return to work in the study period. A prospective follow-up study was conducted of 60 patients with complex fractures of the tibia treated with ring external fixation. Patient-reported outcomes, radiological outcomes and socio-economic status including employment status of the patients were obtained 12 months after frame removal. Forty-six patients completed the assessment 12 months after frame removal (77%). The mean age of the patient at the time of fracture was 54.6 years (range 31–86). There were 19 males and 27 females. At 12 months after frame removal, the mean EQ5D-5L index was 0.66 (CI 0.60–0.72). The mean EQ5D-5L VAS was 69 (CI 61–76). When this was compared to the established reference population from Denmark, the study population showed a significantly worse EQ5D-5L index. The majority of patients (87%) were in the lower social classes suggesting a higher degree of social deprivation in the study population. Twenty-seven per cent of patients who were employed prior to injury had returned to employment at approximately 19 months following fracture. The onset of post-traumatic osteoarthritis was present in the knee joint in 29% of patients following a proximal intra-articular fracture, whereas osteoarthritis was present at the ankle joint in 35% of patients following a distal intra-articular fracture 12 months after frame removal. This study indicates that at 12 months after frame removal there are poorer patient-reported QOL as when compared to reference populations. Furthermore, this study suggests that complex tibial fractures are associated with lower social classes and that only 27% of patients in this sample, who prior to injury were employed, had returned to employment at approximately 19 months after the injury.

## Introduction

The treatment of complex fractures of the tibia, which is defined as involving either the knee or ankle joint surfaces, multi-fragmented shaft fractures or those with major soft tissue damage, is challenging [1, 2]. The long-term outcomes are associated with a risk of knee pain, malalignment, persistent articular damage and an increased risk of post-traumatic osteoarthritis [1, 3–13].

The operative treatment of such injuries is difficult due to the presence of multiple fragments. For joint injuries, the objective of treatment is anatomical reduction, restoration of axial alignment and stable fixation until union [1, 2]. The literature describes several treatment methods—involving external fixation, staged treatment and internal fixation—all with mixed results [1, 3–13]. A number of reports have reported favourable results of the treatment with a ring fixator [3, 4, 10, 12–15], and this is the preferred method of the authors. Prospective studies evaluating patient-reported outcomes, function and development of osteoarthritis following such fractures treated with ring external fixation are lacking.

Axial malalignment and articular injuries of the knee and ankle may increase the risk of post-traumatic osteoarthritis which, in turn, leads to a total knee replacement or fusion of the ankle joint at an early age [7]. Consequently, complex articular fractures of the tibia may produce a significant socio-economic influence on the patient group and lead to a decreased quality of life (QOL) [7].

The primary aim of this study was to report the patient-reported health-related quality of life (QOL) at 12 months after frame removal following a complex fracture of the tibia. The secondary aim was to describe the socio-economic characteristics of the patient sample and report the rate of return to work 12 months after frame removal.

The hypothesis was that patients would report a worse outcome compared with the Danish reference population on EQ5D-5L index score at 12 months after frame removal after a complex tibial fracture.

## Patients and methods

### Study design

This was a prospective study of all patients with a complex tibial fracture treated with a ring external fixator. This study reports the outcomes 12 months after frame removal. The Danish Data Protection Agency (J. nr. 2008-58-0028) approved the study.

The primary outcome measurement was the EQ5D-5L index [16]. The secondary outcomes were patient-reported questionnaires (KOOS, OMAS, MDI), radiological outcomes and the sample’s social class and employment status.

**Fig. 1:**
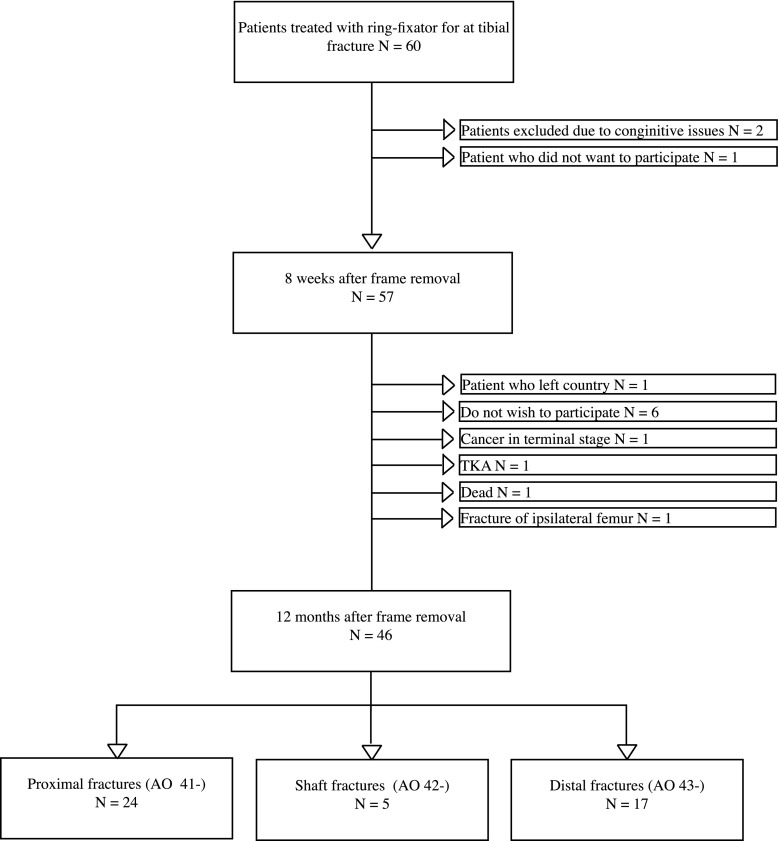
Detailed overview of the study population

All patients were treated with a ring external fixator for a complex fracture of the tibia at Aalborg University Hospital, Denmark, between December 2012 and May 2014. These patients were included in the Trauma Ilizarov Database. Patients with complex tibial fractures not treated with a ring external fixator were excluded. Patients who were unable to fill out the questionnaires due to physical or mental disabilities were excluded. A detailed overview of patient demographics is shown in Fig. 1.

Information about age, gender, fracture classification, co-morbidities and socio-economic data was registered. Fracture classification was performed according to the AO classification [17] and was conducted on the preoperatively obtained CT scan.

### Surgical treatment

Bicondylar fractures of the tibial bone, complex fractures with soft tissue damage of the tibial shaft and distal fractures of the tibial bone not treatable safely by intramedullary nailing were all treated by a ring external fixator. The authors preferred to manage proximal and distal intra-articular tibial fractures with initial screw fixation of articular bone fragments and, if necessary, with exposure of the joint surface. Both autogenous bone grafting and allogenic bone grafting were used. The metaphyseal–diaphyseal junctions of the fracture were bridged by the circular frame. The frame was attached to the bone by hydroxyapatite-coated half pins and Kirschner wires with olives. After applying the ring, external fixator alignment was assessed and corrected. Amendments such as footplates and proximal fixation of the femur were used when deemed appropriate.

### Outcome measurements 12 months after frame removal

#### Patient-reported measurements

EQ5D-5L is a standardized and validated instrument to assess health outcome [16]. It consists of five dimensions: mobility, self-care, usual activities, pain or discomfort, anxiety or depression, and a self-rated health scale on a 20-cm vertical, visual analogue scale with endpoints labelled ‘the best health you can imagine’ and ‘the worst health you can imagine’. Each dimension has five levels: no problems, slight problems, moderate problems, severe problems and extreme problems. A Danish data set was used to calculate the EQ5D-5L index [18]. An EQ5D-5L index of 1.0 indicates full health and 0.0 denotes death. A reference population from Denmark is available [19].

The Knee Injury and Osteoarthritis Outcome Score (KOOS) [20] is a standardized and validated instrument used to evaluate knees and associated problems. The questionnaire includes 42 items, and each item obtains a score from 0 to 4; a total score from 0 to 100 is calculated for each subscale. A total score of 100 indicates no symptoms and 0 indicates major symptoms. KOOS reference data [21] from a general population-based sample in southern Sweden are available.

The Olerud–Molander Ankle Score (OMAS) [22] is a standardized and validated instrument used to evaluate ankle and associated problems. The OMAS is a patient-reported questionnaire, developed to evaluate function after ankle fracture. The scale is a functional rating scale from 0 (totally impaired) to 100 (completely unimpaired) and is based on nine different items: pain, stiffness, swelling, stair climbing, running, jumping, squatting, supports and activities of daily living.

The Major Depression Inventory (MDI) score [23] is a validated system designed to measure depression symptoms in accordance with the symptom guidelines defined by the WHO classification for unipolar depression (ICD-10) and the American Psychiatric Association classification for major depression (DSM-IV). The instrument consists of 12 questions. On a six-point Likert scale, the individual items measure how much of the time the symptoms have been present during the last 14 days. The MDI was scored according to specific guidelines. A score of zero indicates no depression and 50, severe depression. The following categories were used: no depression, less than 20, mild, 20–24, moderate, 25–29 and severe depression, 30 or more, were used [23, 24].

Pain was assessed on a visual analogue scale (VAS) ranging from 0 to 10 cm. Patients were asked to classify pain while resting.

### Socio-economic evaluation and return to work

The patients’ social class was recorded based on a method developed by the Danish National Institute of Social Research [25]. The patients were grouped in five social classes according to three criteria: occupation, education and number of subordinates. The classes were defined as follows: (1) consists of university graduates, self-employed individuals with more than 20 employees, and salaried employees with more than 50 subordinates, (2) consists of self-employed individuals with 6–20 employees and salaried employees with 11–50 subordinates or a medium-long education, (3) consists of self-employed individuals with a minimum of five employees and salaried employees with 1–10 subordinates or with specialized work, (4) includes salaried employees and lower-level and skilled manual workers and (5) consists of unskilled manual workers [25].

Socio-economic evaluation is presented with a comparison of the patient’s pre-injury employment status and employment status 12 months after frame removal. The outcomes included return to work defined as: (1) returned to pre-injury work, (2) returned to work with reduced time, (3) not able to return to work and (4) retired before injury. Data were collected through an interview at the time of admission to the hospital and at follow-up 12 months after frame removal.

#### Radiological outcome measurements

Radiographic examination, including X-rays and preoperative CT scans were obtained from all patients. Post-operatively, X-rays of the entire lower leg were obtained and used to evaluate the quality of reduction. Radiological examination was performed 12 months after frame removal. Intra-articular fractures of the proximal tibia were evaluated for alignment, residual depression of the articular surface and condylar widening as described by Rasmussen et al. [26]. Shaft fractures were evaluated for alignment. Distal intra-articular fractures were evaluated for alignment, talar subluxation, residual central depression and mortise widening as described by Ramos et al. [4]. Furthermore, an assessment of the quality of reduction in distal fractures was performed as described by Marsh et al. [27], modified by Burwell and Charnley [28]. Osteoarthritis was evaluated as described by Kellgren and Lawrence [29]. X-rays were obtained non-weight bearing 6 weeks post-operatively and with weight bearing at follow-up 12 months after frame removal. Radiological outcomes were assessed by a single author (RE).

The authors have evaluated the patient-reported satisfaction during the treatment period of ring external fixation in this same patient group previously. This study presents an extension of that study on the same sample.

### Statistics

The distribution of variables was checked visually for normality by QQ plots. Continuous data were expressed as mean and standard deviation (SD). Categorical data were expressed as frequencies. The statistical analysis was performed by Stata (version 13).

## Results

Sixty patients were treated with a ring external fixator for a complex fracture of the tibia during the study period. A total of 46 patients completed the examination 12 months after frame removal (77%) and were included in the present study. All fractures united during the study period. Fourteen patients were lost to follow-up. A detailed overview of the patients entering the study is presented in Fig. 1.

Twenty-four patients were treated after a proximal tibial plateau fracture, five patients following a diaphyseal fracture and 17 patients following an intra-articular distal tibial fracture. The mean age at the time of fracture was 54.6 years, with a range of 31–86 years. Baseline variables of all patients are presented in Table 1.

**Table 1:** Baseline characteristics

Follow-up time from injury, months (SD)	19.3 (3.3)
Follow-up time from frame removal, months (SD)	12.7 (3.3)
Age at time of fracture, mean (range)	54.6 (31–86)
Gender male/female	19/27
BMI, mean (SD)	25.6 (5.1)
Smoker yes/no	28/18
Side of injury, right/left/bilateral	22/22/2
High-/low-energy trauma	16/30
Poly-/mono-trauma	11/35
Co-morbidities	
ASA score, mean (SD)	1.7 (0.6)
Charlston co-morbidity score, mean (SD)	2.8 (1.7)
Diabetes mellitus	6
*Fracture classification (AO)*	
AO-41	24
A	2
B	0
C	22
AO-42	5
A	3
B	0
C	2
AO-43	17
A	5
B	6
C	6
Open/closed fracture	4/42
AO-41	1
AO-42	1
AO-43	2

AO classification [17]

*SD* standard deviation

### Patient-reported quality of life

Twelve months after frame removal, the mean EQ5D-5L index was 0.66 (SD 0.19). The mean EQ5D-5L VAS was 69 (SD 24.4). Compared with the established reference population from Denmark [19], the study population showed a significantly worse EQ5D-5L index. The EQ5D-5L scores divided into AO type 41, 42 and 43 are presented in Table 2.

**Table 2:** Patient-reported outcomes 12 months after frame removal compared with reference populations

	KOOS				
	PAIN	ADL	SYMP	QOL	SPORT
*Proximal fracture (AO 41-)*					
Study population					
Mean	68.9	71.4	64.9	52.1	30.4
95% CI	58.5–79.2*	62.2–80.7*	54.3–75.4*	39.0–65.3*	19.2–41.6*
Reference population**,***					
95% CI	86.7–88.2	86.5–88.1	85.4–86.9	77.4–79.6	72.5–75.1

	EQ5D-5L	
	Index	VAS
Study population		
Mean	0.715	76
95% CI	0.635–0.795*	67.5–84.4
Reference population**** (male/female 50–59 years)		
Mean	0.888/0.858	
95% CI	0.880–0.896/0.850–0.866	

	EQ5D-5L	
	Index	VAS
*Shaft fracture (AO 42-)*		
Study population		
Mean	0.681	56
95% CI	0.576–0.786*	27.8–84.2
Reference population**** (male/female 50–59 years)		
Mean	0.888/0.858	
95% CI	0.880–0.896/0.850–0.866	

	EQ5D-5L	
	Index	VAS
*Distal fracture (AO 43-)*		
Study population		
Mean	0.591	63.7
95% CI	0.476–0.706*	48.5–79.0
Reference population**** (male/female 50–59 years)		
Mean	0.888/0.858	
95% CI	0.880–0.896/0.850–0.866	

* Significantly different compared with reference population

** Paradowski et al. [21]

*** Unpublished data. Ewa Roos ‘Personal communication’ 13 November 2012. Paradowski et al. [21]

**** Sorensen et al. [19]

### Socio-economic evaluation and return to work

The distribution of social classes at the time of admission to hospital showed three patients in group I, three patients in group III, 22 patients in group IV and 18 patients in group V. The majority (87%) of patients were grouped into social classes IV and V, indicating a high degree of social deprivation in the study population.

Thirty-four of the 46 patients in the study population were below the age of 65 years, which was the official retirement age in Denmark in 2014. Twenty of these 34 patients were employed prior to the occurrence of the injury, and two patients above the age of 65 years were employed. Of the 22 patients who were employed prior to the injury, six patients returned to pre-injury work, nine patients were employed with reduced working hours, and seven patients were unable to return to work, 12 months after frame removal. Both patients above the age of 65 years returned to employment.

### Indication of depression

Twelve months after frame removal, the MDI score, indicating psychological status, showed 41 patients reporting no depression. Four patients reported MDI scores between 20 and 30, indicating mild-to-moderate depression, and one patient reported a score of more than 30, indicating severe depression.

### Tibial plateau fracture (AO 41-): 24 patients

Twelve months after frame removal, the mean KOOS score were pain, 69 (SD 24); symptoms, 65 (SD 26); ADL, 71 (SD 24); sport, 30 (SD 29); and QOL 52 (SD 32). Compared with the established reference population [21], the study population showed a significantly worse KOOS outcome for all five subgroups. See Table 2.

The VAS score for resting pain was reported as a range from zero to six with an average of 1.3 (SD 2.1). Sixteen patients reported no pain at rest, five patients reported a VAS between one and three, and three patients reported a VAS scores between four and six.

#### Radiological outcomes

Five of the 24 patients had either malalignment, condylar widening more than 5 mm and/or articular depression of more than 5 mm 12 months after frame removal. A detailed overview is presented in Table 3. The radiological outcomes of osteoarthritis of the knee (Kellgren and Lawrence) show three patients with no or doubtful signs of osteoarthritis (Type 0 and Type 1), 13 patients with minimal signs of osteoarthritis (Type 2) and six patients with moderate signs of osteoarthritis (Type 3). One patient with Type 3 osteoarthritis was treated with a total knee arthroplasty (TKA) during the study period.

**Table 3:** Radiological malalignment

Proximal (41)	
Malalignment > 3°	2
Condylar widening > 5 mm	2
Depression > 5 mm	3
Number of affected patients	5
Shaft (42)	
Malalignment > 3°	1
Distal (43)	
Malalignment > 3°	1
Central Depression > 5 mm	2
Number of affected patients	3

12 months after frame removal, the radiological assessments were made on AP and side X-rays. Proximal tibial fractures were evaluated concerning alignment and depression of the articular surface and condylar widening as described by Rasmussen et al. [27]. Shaft fractures were evaluated concerning alignment. Distal fractures were evaluated with regard to alignment, talar subluxation, central depression and mortise widening as described by Ramos et al. [4]

### Tibial diaphyseal fracture (AO-42): five patients

The VAS score for resting pain was reported as a range from 0 to 7 with an average of 1.4 (SD 3.1). Four patients reported no pain at rest and one patient reported a VAS score of seven.

#### Radiological outcomes

One of the five patients treated for a shaft fracture had a varus deformity of six degrees. The radiological outcomes for osteoarthritis of the ankle and knee (Kellgren and Lawrence) showed five patients with none or doubtful signs of osteoarthritis (Type 0 and 1).

### Fracture of the distal tibia (AO 43-): 17 patients

The mean Olerud–Molander Ankle Score 12 months after frame removal was 43(SD 32). No reference population was available for the Olerud–Molander Ankle Score.

The VAS score for resting pain was reported as a range from zero to eight with an average of 1.0 (SD 2.4). Thirteen patients reported no pain at rest, two patients reported a VAS between one and three, one patients reported a VAS of five, and one patient reported a VAS score of eight.

#### Radiological outcomes

Three of the 17 patients treated for a distal intra-articular fracture had either malalignment of more than 3 degrees or a central depression of more than 5 mm. A detailed overview is presented in Table 3. The radiological outcomes of osteoarthritis of the ankle (Kellgren and Lawrence) showed seven patients with no or doubtful signs of osteoarthritis (Type 0 and 1), four patients with minimal signs of osteoarthritis (Type 2), four patients with moderate signs of osteoarthritis (Type 3) and two patients with severe signs of osteoarthritis (Type 4).

These two patients with Type 4 osteoarthritis were treated with a talocrural fusion (11 and 32 months following primary injury, respectively).

## Discussion

The treatment of these fractures of the tibia is challenging due to the multi-fragmented nature and the involvement of joint surfaces and concomitant soft tissue injuries. Moreover, the fractures are often a result of high-energy trauma [3, 4] and significant complications during treatment are common [2, 30, 31].

This study shows a considerable social deprivation of the study population with 87% of the population in the lower social classes (IV and V) compared with the general population in Denmark with 46% in the lower social classes [32]. This increased incidence of complex tibial fractures in the lower social classes with an increased likelihood of deprivation is supported by Court-Brown et al. [33] who reported a significant increase in the incidence of fractures in the most deprived 10% of the population with most fracture types affected. Whether this increased risk is based on behavioural differences, is work related or is due to economic differences or other factors, is not well established.

Seventy-four per cent of the study population was below the retirement age of 65 years. Fifty-nine per cent of the patients below 65 years of age were employed prior to injury. There was a substantially higher degree of unemployment in the study group compared with the national Danish general unemployment rate of less than 5% [34]. Twelve months after frame removal, 27% of the patients who had been employed prior to the fracture had returned to work. This suggests a high risk of attrition from employment in the study population after a complex fracture of the tibia. This might be due to the high proportion of lower social classes in the sample which are often employed in manual labour. This type of employment might indicate an increased dependency on a high level of function of the lower extremity. Furthermore, as part of the Danish flexicurity model, Denmark has a high level of income benefit constituting upwards of 90% [40] of the lowest salaries initially, and this may influence the rate of employment of those with disability.

Taking individual patient information into account when planning post-operative treatment methods may be important. Development of special rehabilitation algorithms directed towards this patient group including social and work-related interventions may improve outcome. However, more research is needed to combine social and health science.

The patient-reported outcomes 12 months after frame removal showed results below the established reference populations [19, 21] in both generic (EQ5D-5L) and symptom-specific questionnaires (KOOS). Studies reporting on patient-reported outcome following complex fractures of the tibia support the persistence of limitations in QOL throughout time [3, 4, 15, 35]. However, most studies evaluating patient-reported outcomes do not use a reference population or pre-injury values. A recent study by The Canadian Orthopaedic Trauma Society [15] indicated that patient-reported outcomes are more sensitive to residual disability than radiological evaluations. Furthermore, it is documented that low levels of education are associated with a worse QOL [19].

The onset of post-traumatic osteoarthritis following fractures of the tibia involving the joint surfaces has been the subject of several studies. The incidence of osteoarthritis following tibial condyle fractures is reported to be between 17 and 83% with a wide range in the severity of osteoarthritis [1, 36]. In the present study, 29% of patients with proximal tibial plateau fractures demonstrated moderate-to-severe osteoarthritis of the knee at a mean of 19.3 months follow-up after fracture. These findings are in line with other studies reporting osteoarthritis following tibial plateau fractures [7, 15].

Moreover, early onset of osteoarthritis is reported in distal fractures involving the ankle joint [4, 37]. In the present study, 35% of the patients in the group with distal fractures demonstrated moderate-to-severe signs of osteoarthritis at follow-up, which is comparable to previous studies reporting on osteoarthritis following distal tibial fractures [37].

This study has several limitations. First, this is a prospective follow-up study which precludes the drawing of conclusions regarding causality. Another limitation of this study is the number of patients included, which limits comparisons or other associations between the groups. Furthermore, the study is limited by a minimum follow-up time of 12 months after frame removal. Other studies have shown that the development of post-traumatic osteoarthritis is affected by the time after the fracture [38, 39]. Regarding the social classes and return to previous employment, this study evaluates patients in a country with a high degree of social insurance including a high level of publicly financed unemployment insurance. This may lead to a lower level of incentive to return to work as compared with other countries.

## Conclusion

This study shows that patients 12 months after frame removal report significantly worse patient-reported QOL as compared to reference populations. Furthermore, this study demonstrates that complex tibial fractures are associated with lower social classes and that 27% of patients, who prior to injury were employed, returned to employment at approximately 19 months after the fracture.
